# First Insights into the Genome of *Fructobacillus* sp. EFB-N1, Isolated from Honey Bee Larva Infected with European Foulbrood

**DOI:** 10.1128/genomeA.00868-15

**Published:** 2015-07-30

**Authors:** Marvin Djukic, Rolf Daniel, Anja Poehlein

**Affiliations:** Genomic and Applied Microbiology and Göttingen Genomics Laboratory, Institute of Microbiology and Genetics, Georg-August University Göttingen, Göttingen, Germany

## Abstract

European foulbrood is a worldwide disease affecting the honey bee brood. Here, we report the draft genome sequence of *Fructobacillus* sp. EFB-N1, which was isolated from an infected honey bee larva derived from a Swiss European foulbrood outbreak. The genome consists of 68 contigs and harbors 1,629 predicted protein-encoding genes.

## GENOME ANNOUNCEMENT

*Fructobacillus* sp. EFB-N1 was isolated from a honey bee larva infected with European foulbrood (EFB). The nonmotile isolate grows aerobically on de Man/Rogosa/Sharpe (MRS) agar (1) at 32°C. The 16S rRNA gene sequence comparison showed a 99.7% DNA sequence identity to *Fructobacillus tropaeoli* F214-1, which is a fructophilic lactic acid bacterium isolated from a flower (2). Comparison of *recA* gene sequences is used as a marker for classification of *Fructobacillus* species (3, 4). The *recA* gene sequence of strain EFB-N1 revealed 91% DNA sequence similarity to the corresponding gene of *F. tropaeoli*.

Chromosomal DNA of *Fructobacillus* sp. EFB-N1 was isolated with the MasterPure complete DNA purification kit as recommended by the manufacturer (Epicentre, Madison, WI, USA). The extracted DNA was used to generate Illumina shotgun libraries, which were subsequently sequenced with a Genome Analyzer IIx as recommended by the manufacturer (Illumina, San Diego, CA, USA). Trimming and removal of low-quality reads with Trim Galore version 0.4.0 (http://www.bioinformatics.babraham.ac.uk/projects/trim_galore) resulted in 5,374,914 paired-end Illumina reads. Genome assembly was performed with SPAdes software version 3.5.0 (5) and yielded 68 contigs (>500 bp) and 368-fold coverage. The draft genome of *Fructobacillus* sp. EFB-N1 comprises 1.64 Mbp, with an overall G+C content of 43.7%. Open reading frame (ORF) and RNA detection were verified with Prodigal version 2.6.2 (6) and Barrnap version 0.6 (http://www.vicbioinformatics.com/software.barrnap.shtml), respectively. Annotation was performed with Prokka version 1.11 (7). The draft genome harbored one rRNA cluster, 46 tRNA genes, 1,210 predicted protein-coding genes with function assignment, and 419 putative genes coding for hypothetical proteins.

Genes coding for a putative bacterial conjugation machinery were located on contig FEFB_c000008. In addition, potential genes encoding proteins involved in adhesion and biofilm formation that showed high amino acid sequence similarity to corresponding predicted proteins encoded by the genomes of other Fructobacilli were identified. Noteworthy, the genome of *Fructobacillus* sp. EFB-N1 lacks a complete glycolysis system and pentose phosphate pathway, as putative genes encoding phosphofructokinase, fructose-bisphosphate aldolase, transketolase, and transaldolase were missing. Additionally, strain EFB-N1 completely lacked genes coding for citrate cycle enzymes. A putative lactate dehydrogenase-encoding gene as part of a homolactic fermentation pathway is present. In addition, the presence of a putative sucrase-encoding gene indicated the ability to convert sucrose to fructose and glucose.

### Nucleotide sequence accession numbers.

This whole-genome shotgun project has been deposited at DDBJ/EMBL/GenBank under the accession number LDUY00000000. The version described in this paper is the first version, LDUY01000000.

## References

[1] De Man JC, Rogosa M, Sharpe ME 1960 A medium for the cultivation of lactobacilli. J Appl Bacteriol 23:130–135. doi:10.1111/j.1365-2672.1960.tb00188.x.

[2] Endo A, Irisawa T, Futagawa-Endo Y, Sonomoto K, Itoh K, Takano K, Okada S, Dicks LM 2011 *Fructobacillus tropaeoli* sp. nov., a fructophilic lactic acid bacterium isolated from a flower. Int J Syst Evol Microbiol 61:898–902. doi:10.1099/ijs.0.023838-0. 20495031

[3] Torriani S, Felis GE, Dellaglio F 2001 Differentiation of *Lactobacillus plantarum*, *L. pentosus*, and *L*. *paraplantarum* by *recA* gene sequence analysis and multiplex PCR assay with *recA* gene-derived primers. Appl Environ Microbiol 67:3450–3454. doi:10.1128/AEM.67.8.3450-3454.2001. 11472918 PMC93042

[4] Endo A, Okada S 2008 Reclassification of the genus *Leuconostoc* and proposals of *Fructobacillus fructosus* gen. nov., comb. nov., *Fructobacillus durionis* comb. nov., *Fructobacillus ficulneus* comb. nov. and *Fructobacillus pseudoficulneus* comb. nov. Int J Syst Evol Microbiol 58:2195–2205. doi:10.1099/ijs.0.65609-0. 18768629

[5] Bankevich A, Nurk S, Antipov D, Gurevich AA, Dvorkin M, Kulikov AS, Lesin VM, Nikolenko SI, Pham S, Prjibelski AD, Pyshkin AV, Sirotkin AV, Vyahhi N, Tesler G, Alekseyev MA, Pevzner PA 2012 SPAdes: a new genome assembly algorithm and its applications to single-cell sequencing. J Comput Biol 19:455–477. doi:10.1089/cmb.2012.0021. 22506599 PMC3342519

[6] Hyatt D, Chen G-L, Locascio PF, Land ML, Larimer FW, Hauser LJ 2010 Prodigal: prokaryotic gene recognition and translation initiation site identification. BMC Bioinformatics 11:119. doi:10.1186/1471-2105-11-119. 20211023 PMC2848648

[7] Seemann T 2014 Prokka: rapid prokaryotic genome annotation. Bioinformatics 30:2068–2069. doi:10.1093/bioinformatics/btu153. 24642063

